# Thermal desorption treatment of petroleum hydrocarbon-contaminated soils of tundra, taiga, and forest steppe landscapes

**DOI:** 10.1007/s10653-020-00802-0

**Published:** 2021-01-16

**Authors:** Marina V. Bykova, Alexey V. Alekseenko, Mariya A. Pashkevich, Carsten Drebenstedt

**Affiliations:** 1 Department of Geoecology, Saint Petersburg Mining University, 2, 21st line V.O., Saint Petersburg, Russian Federation 199106; 2 Technische Universität Bergakademie Freiberg, 1a, Gustav-Zeuner-Str., Freiberg, 09596 Germany

**Keywords:** Spills and leaks, Oil-contaminated soils, Environmental impact assessment, Ex situ treatment, Pyrolytic remediation, Organic carbon, Soil fertility, Land restoration, Brownfield revitalization

## Abstract

The results of field, analytical, and experimental research at a number of production facilities reflect the properties of oil-contaminated soils in 3 landscapes: the permafrost treeless Arctic ecosystem, boreal forest, and temperate-climate grassland-woodland ecotone. Laboratory studies have revealed the concentrations of petroleum hydrocarbons in soils, ranging from medium levels of 2000–3000 mg/kg to critical figures over 5000 mg/kg, being 2–25 times higher than the permissible content of oil products in soils. The experimentally applied thermal effects for the oil products desorption from the soil allowed finding an optimal regime: the treatment temperature from 25 to 250 °C reduces the concentrations to an acceptable value. The conditions are environmentally sound, given that the complete combustion point of humates is ca. 450 °C. The outcomes suggest the eco-friendly solution for soil remediation, preserving the soil fertility in fragile cold environments and in more resilient temperate climates, where revitalized brownfields are essential for food production.

## Introduction

### Sources of soil contamination with petroleum products

In the modern era, petroleum hydrocarbons are applied ubiquitously, which consequently results in a severe issue of soil contamination. Emergency spills and technological leaks occur throughout the entire life cycle of oil products. Sources of soil contamination with petrochemicals include all production facilities where they are used for various purposes. Soils are principally affected in three groups of technological areas: oil field development, transportation of hydrocarbons, and industrial enterprises.

Complex crude oil extraction facilities are separated geographically but connected via pipelines, power transmission networks, and transport systems (Aleksandrova et al. 2017; Dolgii 2018; Khormali 2017; Khormali et al. 2018; Kondrasheva et al. 2017). Occupying vast territories, the production facilities of oil fields exercise a significant effect on the components of the environment and are a potential source of man-made pollution flows (Shuvalov et al. 2008). It is a known fact that soil contamination with petroleum products occurs properly during drilling and production of hydrocarbons, while, in addition to on-land plants, marine production is a serious threat to the soil due to pollution of coastal areas (Cozzarelli et al. 2017; Berkadu et al. 2018; Correa Pabón et al. 2019). According to certain findings, operational issues of extraction equipment are one of the most common causes of emergency spills (Clancy et al. 2018).

Localization patterns of soil contamination with petroleum products in the areas of transportation are determined by the presence of main pipeline systems. Their long length causes a high risk of accidental spills (Balseiro-Romero et al. 2018; Liang et al. 2018; Liu et al. 2019).

Areas of industrial enterprises represent the most diverse and numerous group of petrochemical sources and pathways to the environment, which includes territories of processing, storage, and usage of petroleum products (Golubev and Karpova 2017; Iakovleva et al. 2017; Koptev and Kopteva 2017; Privalov and Privalova 2017; Sobota et al. 2019; Zyrin and Ilinova 2016). Production facilities in the oil processing areas are a source of condensate and lubricants, as well as various chemical reagents (Kondrasheva et al. 2019; Nikitin and Saychenko 2018; Nikolaevna and Sergeevich 2020; Pivovarova and Makhovikov 2017; Shuvalov et al. 2008; Struchkov and Rogachev 2018). When storing petroleum products, soil contamination occurs due to frequent leaks from reservoirs or non-compliance with pumping processes (Lee et al. 2019; Gorawski et al. 2017; Sacile 2007). When storing viscous petrochemicals, sediments can accumulate due to incompatibility of petroleum products, which can affect the corrosion of the metal, the stress–strain state of the reservoir structure, and accident rate resulting in the ingress of petroleum products into the soil (Sultanbekov et al., 2019a, 2019b,^ 2020^). It is found that industrial facilities such as gas stations, despite the relatively small taken area, contribute significantly to the contamination of adjacent soils with petroleum products that also occurs due to leaks from fuel storage tanks (Aulia et al. 2016; Iurchenko et al. 2017).

In the areas where oil products are used, the formation of technogenic flows is caused by high levels of fuel consumption by road and rail transport, as well as by lubricants applied during the equipment operation. This group includes transport mechanic shops, machine-building enterprises, highways, parking houses, etc.

According to the official information from the recent report of *the Russian Federal Service for Ecological, Technological and Nuclear Supervision (Rostekhnadzor)*, the following sites are registered in Russia:

– 7,864 oil and gas production facilities;

– 4,389 objects of the petrochemical, oil, and gas processing industry and oil product supply facilities (oil depot sites for storage and transfer of oil products, fuel and lubricants storage facilities, tanks and unloading systems); and.

– 4,273 objects of major pipeline transport (oil and gas pipelines) and underground gas storages (Annual 2019).

**Fig. 1:**
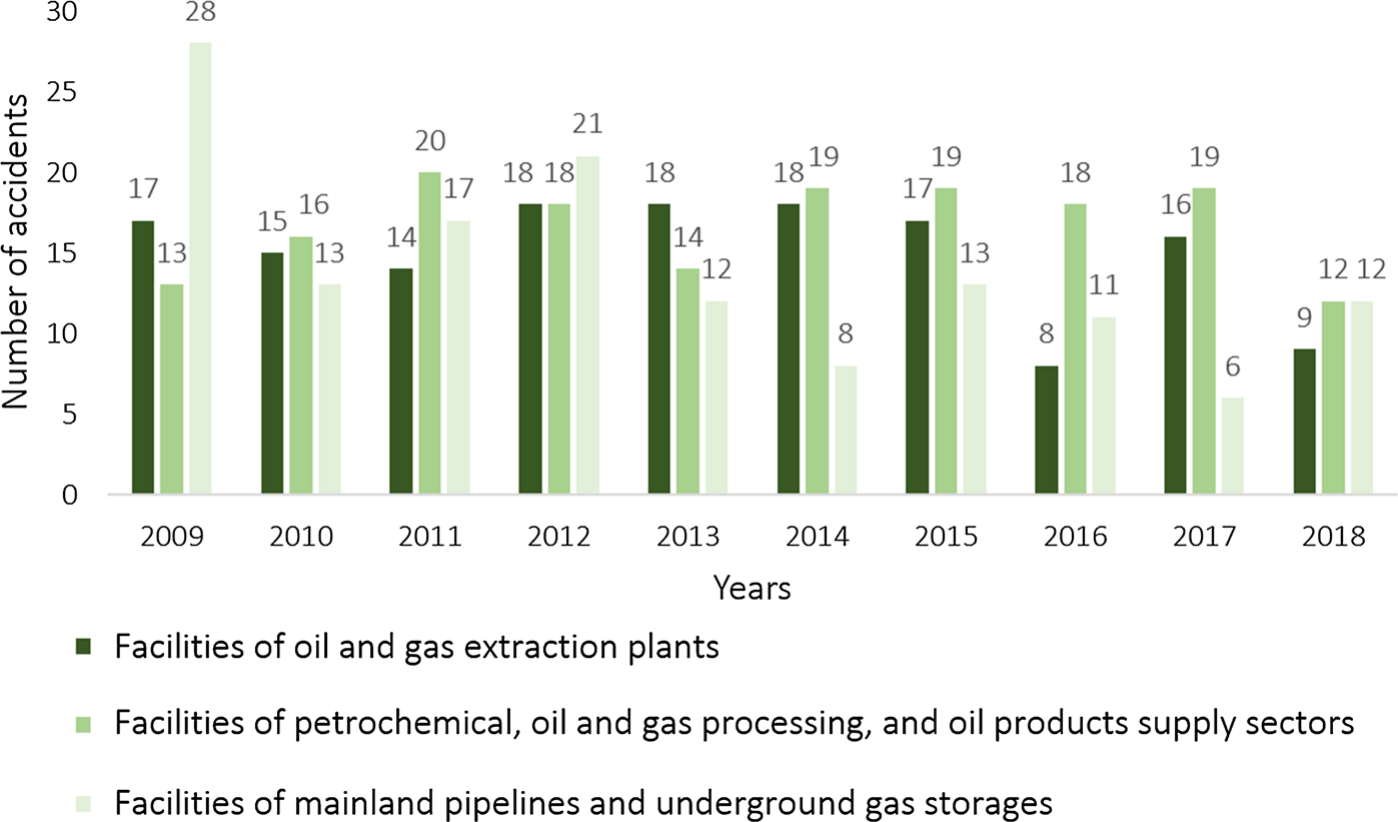
The dynamics of accidents at hazardous production facilities of the oil and gas industry in Russia in 2009–2018 that resulted in spills of oil products (after the Russian Federal Service for Ecological, Technological and Nuclear Supervision)

**Fig. 2:**
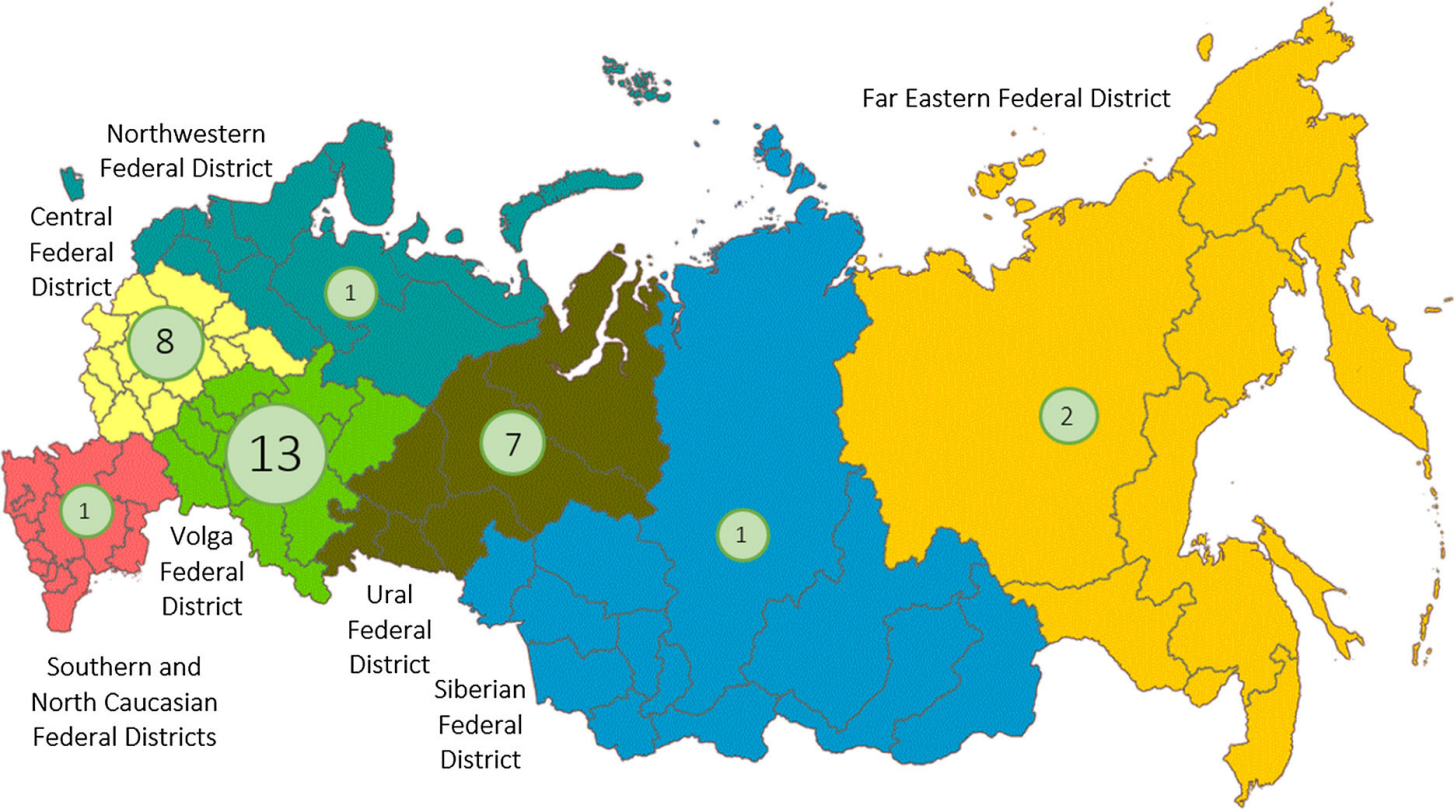
The statistics of major accidents at production facilities of the oil and gas industry in various Federal Districts of Russia in 2018 (after the Russian Federal Service for Ecological, Technological and Nuclear Supervision)

Figure 1 shows the dynamics of accidents at hazardous production facilities of the oil and gas industry in Russia in 2009–2018 that resulted in spills of oil products. Figure 2 provides statistics on the number of accidents in 2018 at production facilities of the oil and gas industry in various Federal Districts of Russia. The information provided reflects the number of major accidents, and the cases at oil rather than gas facilities make the prevailing part (Litvinenko 2020). When it comes to local spills and technological leaks, it is impossible to provide statistics since these incidents are not taken into account.

### Environmental consequences of soil contamination

Entering the soil, petroleum products alter its properties. The humus horizon is experiencing the most serious transformation. The composition of humus is qualitatively and quantitatively changed as the content of technogenic organic carbon increases; this leads to a deterioration of its properties as a nutritious substrate. The redox conditions of soils are changed, and the mobility of humus components is raised. Getting into the soil, oil products increase its hydrophobicity, which disrupts the mechanisms of water supply to the roots of plants. These changes lead to the inhibition of photosynthetic activity and the productivity of plants, which is expressed in their suppression or complete death (Bykova et al. 2019). Accordingly, oil-contaminated soils of agricultural importance are to be remediated on a first-priority basis to prevent the possible total crop failure.

The presence of petroleum products in soils disturbs soil microbiocenoses (Khudur et al. 2019). The outcomes of microbiological studies show that with the increasing oil contamination of soils, there is a decrease in the number of grown colonies of microorganisms that are not able to oxidize hydrocarbons (Merzlyakova et al. 2017; Usacheva 2013). The results of biological tests show that changes in the properties of soils exposed to petroleum products lead to a decrease in the survival rate of numerous soil invertebrates (Kovaleva et al. 2017; Gainer et al. 2019).

The severity of the consequences of soil contamination with petroleum products depends on lots of factors but has the same mechanisms as described above. Diverse ecosystems have their characteristics that can affect the degree of contamination and the ability of soil self-remediation.

Thus, in areas of cold and moderately cold climates where the amount of precipitation exceeds their evaporation (e.g., tundra or taiga landscape zones), a probability of formation of stable emulsions of petrochemicals with water during spills and leaks is higher. This can result in their accumulation and generate lithogeochemical plumes and pollution flows (Alekseenko et al. 2017; Boev et al. 2019; Jordán et al. 2016; Kruk et al. 2018; Sobolev et al. 2018). The humus content in these lands is low and ecosystems are unstable and almost incapable of self-cleaning. The severity of the consequences of oil pollution in the humid natural zones is complicated by high soil humidity and marshiness, which can lead to the migration of oil products with groundwater. Oil-polluted disturbed peat soils become vulnerable to further degradation (Kremcheev 2018). As found by the recent study of Kovaleva et al. (2020), while peat has the ability to absorb some petroleum hydrocarbons, excess oil migrates in soil both downward and laterally. In terms of the current views on peat as a heteroporous system, Selyanina et al. (2019) revealed that the high degree of decomposition results in the destruction and compaction of the capillary-porous structure; this reduces the oil accumulation capacity, which is mainly due to the effect of physical sorption. In the peat soils from West Siberia of Russia, concentrations of petroleum hydrocarbons that will result in 80% functional reductions (i.e., near complete loss on functional activities) are: worm's production—177,000 mg/kg, catalase activity—123,000 mg/kg, and basal respiration—311,000 mg/kg (Kovaleva et al. 2020). Furthermore, the development of a bitumen crust on the peat surface preventing vegetation growth is one of the main issues upon the contamination of peatbog ecosystems, often accompanied by metal accumulation that adversely affects both the natural and artificial destruction of oil by microbes (Vodyanitskii et al. 2012).

In the case of semiarid and arid climate with the evaporation of moisture in a greater amount than rainfall (i.e., steppe, dry steppe, semi-desert, and desert climatic zones), a process of partial evaporation of light oil fractions from the upper layers of contaminated soils becomes possible. Though, most of the heavy petroleum components remain, also forming, in this case, lithogeochemical pollution flows and plumes, being accumulated and/or transported under the action of gravitational force.

Local soil contamination is a high potential hazard since minor spills and leaks are frequently ignored. Long-term spotting of petroleum products in the soil can lead to serious consequences and affect the entire ecosystem (Bykova et al. 2019) that is reflected in the permissible level of petroleum products of 1000 mg/kg adopted in Russia (Table 1).

**Table 1:** Threshold concentrations of petroleum hydrocarbons in soils of the Russian Federation

Contamination level	Petroleum products content, mg/kg
Allowable	< 1000
Low	1000–2000
Medium	2001–3000
High	3001–5000
Critical	> 5000

### Thermal desorption treatment approaches

Treatment of petroleum hydrocarbon contaminated soils can be performed with the help of chemical engineering technologies like the application of persulfate or potassium permanganate (Bajagain et al. 2020; Yen et al. 2011). Application of bioremediation approaches is another option, e.g., by stimulating indigenous microbes, plant-bacteria partnerships, and biodegradation by a microbial consortium or by natural attenuation, biostimulation, and bioaugmentation (Bento et al. 2005; Ghazali et al. 2004; Khan et al. 2013; Liu et al. 2010). Application of chemicals is fraught with challenges and further environmental concerns, while biotechnologies are eco-friendly but slow-acting; it is against this background that we used the thermal desorption as the main mechanism for cleaning soils from petroleum products (Falciglia et al. 2011, 2013, 2014; Jia et al. 2020; Kang et al. 2020; Li et al. 2020; Ren et al. 2020; Yi et al. 2016). We believe that the nature-like technology of thermal treatment that is centered around the process similar to the evaporation in a semiarid and arid climate is the golden mean, fast-operating and environmentally sound.

Thermal desorption technologies are capable of cleaning the soil from various hydrocarbons, but these technologies have not been widely used in Russia. The method has a large number of advantages, such as suitability to various types of pollutants, short treatment time, high efficiency, and safety (Zhao et al. 2019). The process of thermal desorption has even found application in the purification of soils from organochlorine compounds and mercury (Ma et al. 2019; Hou et al. 2016). There are known studies of thermal desorption treatment of drilling mud contaminated with petroleum products in which a decrease in the content of hydrocarbons after treatment was noted (Liu et al. 2019). It is also reported that studies of thermal desorption treatment at a temperature of 380 °C of soils with artificially introduced petroleum products (such as motor oil) were performed for concentrations from 2,000 to 10,000 mg/kg (Kastanek et al. 2016). However, the processing temperature is close to the burning point of all humates (450 °C) and can lead to a significant reduction in soil quality and sintering of soil particles, which will make it difficult to restore an ecosystem.

## Research questions

Since the exploration was aiming at the assessment and abatement of the environmental threat posed by the technogenic soil contamination with petroleum hydrocarbons and the studied areas were situated in different climatic agricultural zones, the major issues had been raised as follows.

– At which contamination levels does remediation remain possible and practicable in the Technosols derived from the Gleysols, Podzols, and Phaeozems (IUSS 2014), in view of the future reuse of the treated soils?

– What is the optimal working temperature range of the ex situ thermal desorption treatment, preserving the fertility of the remediated soils?

## Materials and methods

### Study areas and sampling procedures

**Fig. 3:**
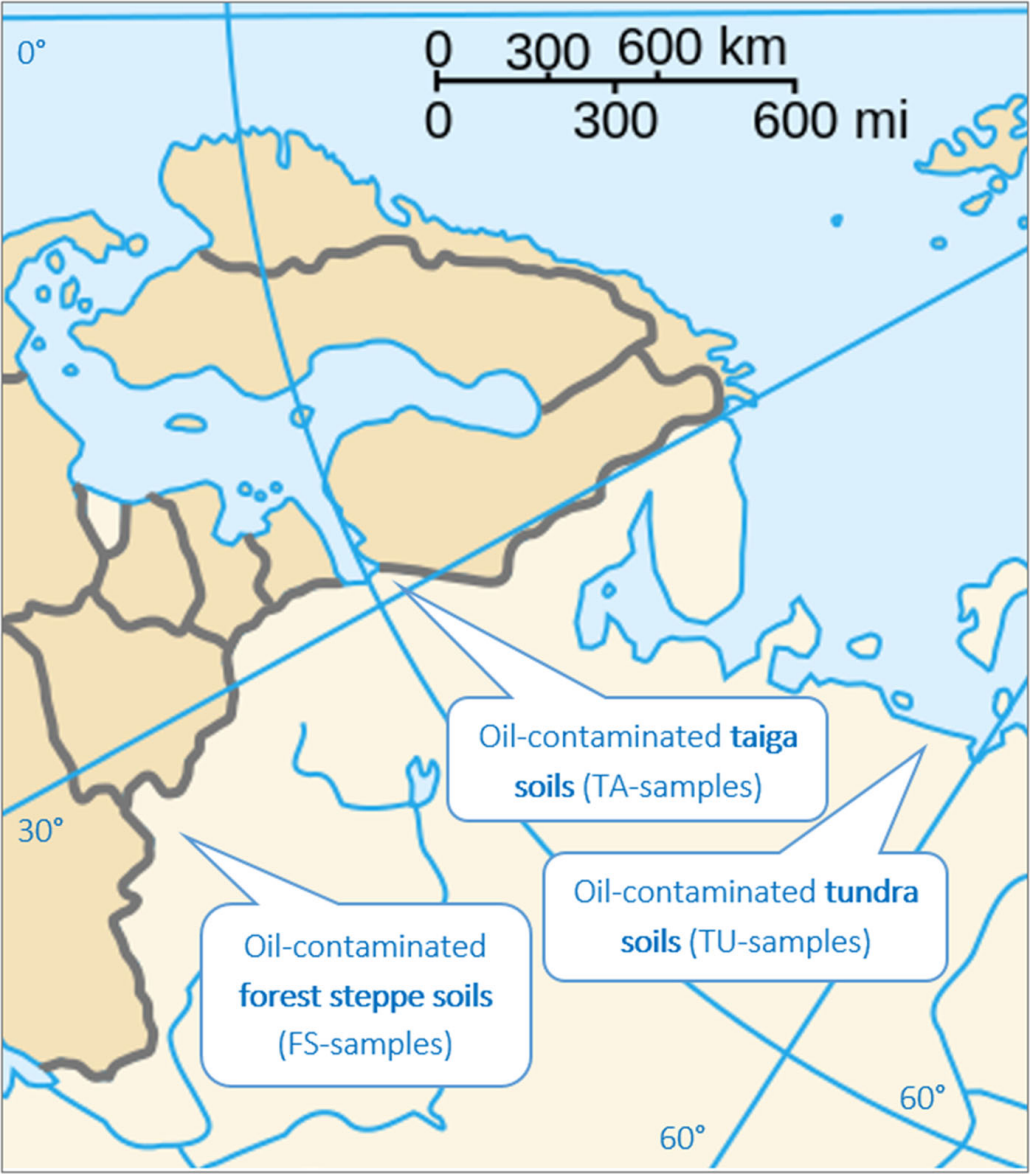
Studied hotspots of petroleum hydrocarbon soil contamination in the tundra, taiga, and forest steppe landscapes (based on the free blank map, *commons.wikimedia.org*)

To assess the degree of soil contamination at various production facilities, a complex investigation was conducted, including field site assessment, sampling, and laboratory studies. At the first stage, sampling sites were determined within the hotspots of petroleum hydrocarbon soil contamination in the tundra, taiga, and forest steppe landscapes (Fig. 3) by one or more independent criteria:

- the study area was partially or completely devoid of vegetation (visual assessment);
- soils had a characteristic smell of petroleum products of different intensity (organoleptic assessment);
- production facilities were in close proximity, which raised the probability of leaks and spills (subjective probabilistic assessment).

A drilling site of a preserved well was *the first study object*. The location of the production facility belongs to the tundra zone of Russia: the climate is humid and cold, and summers are short. The tundra gley soils are cryolithic, swampy, with excessive moisture and insignificant humus content. Depending on the permafrost development, they can be documented internationally as either Cryosols or Gleysols (IUSS 2014). Vegetation is sparsely represented, comprising mainly mosses, lichens, shrubs, and dwarf trees (Naumov 2016). Ecosystems are sensitive to man-made impacts and almost incapable of self-restoration.

**Fig. 4:**
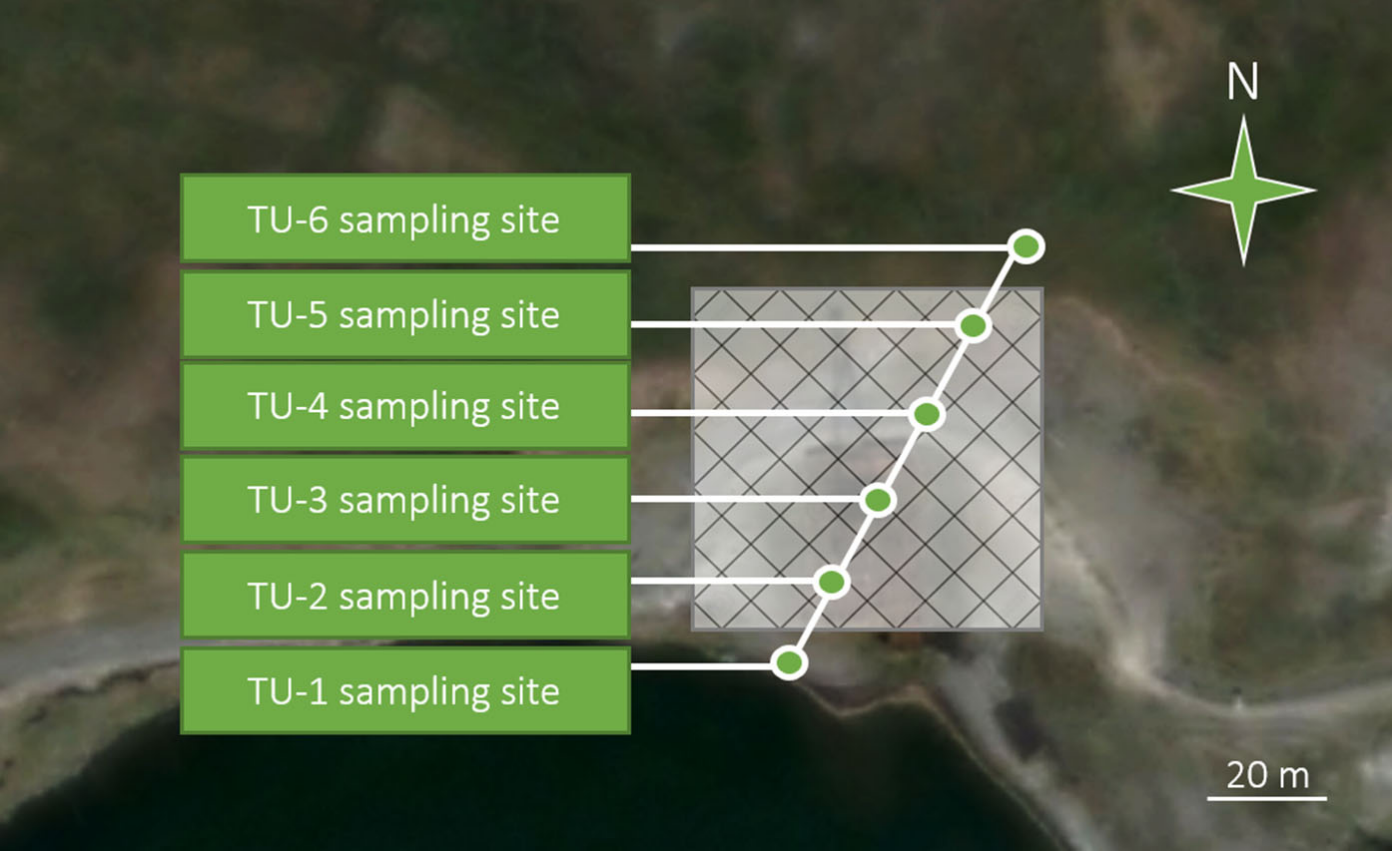
Layout of soil sampling sites in the tundra landscape; the semitransparent diagonal-gridded polygon covers the production site (based on the *Bing Maps*)

Based on the results of the environmental survey conducted in the summer of 2015, a narrow sampling area was selected on the site: a 100-m-long line-shaped soil-geomorphological profile, crossing the drilling site of the well and going beyond the site boundary. In total, 6 test sites of 1 × 1 m were laid within the pedo-geomorphological transect with a 20-m interval between them. The soil sampling scheme is shown in Fig. 4.

A tank farm where petroleum products were transported and stored was *the second study area*. The territory of the production facility belongs to the taiga landscape zone: the climate is moderately cold and humid; soils affected by permafrost can thaw by about a hundred centimeters. This boreal climate sector covers ca. 50% of the Russia's land area. The soils of the investigated site are humid sandy Podzols with a low humus content (IUSS 2014). The vegetation cover is represented by spruce, birch, larch, pine, and fir, as well as mosses (Naumov 2016).

**Fig. 5:**
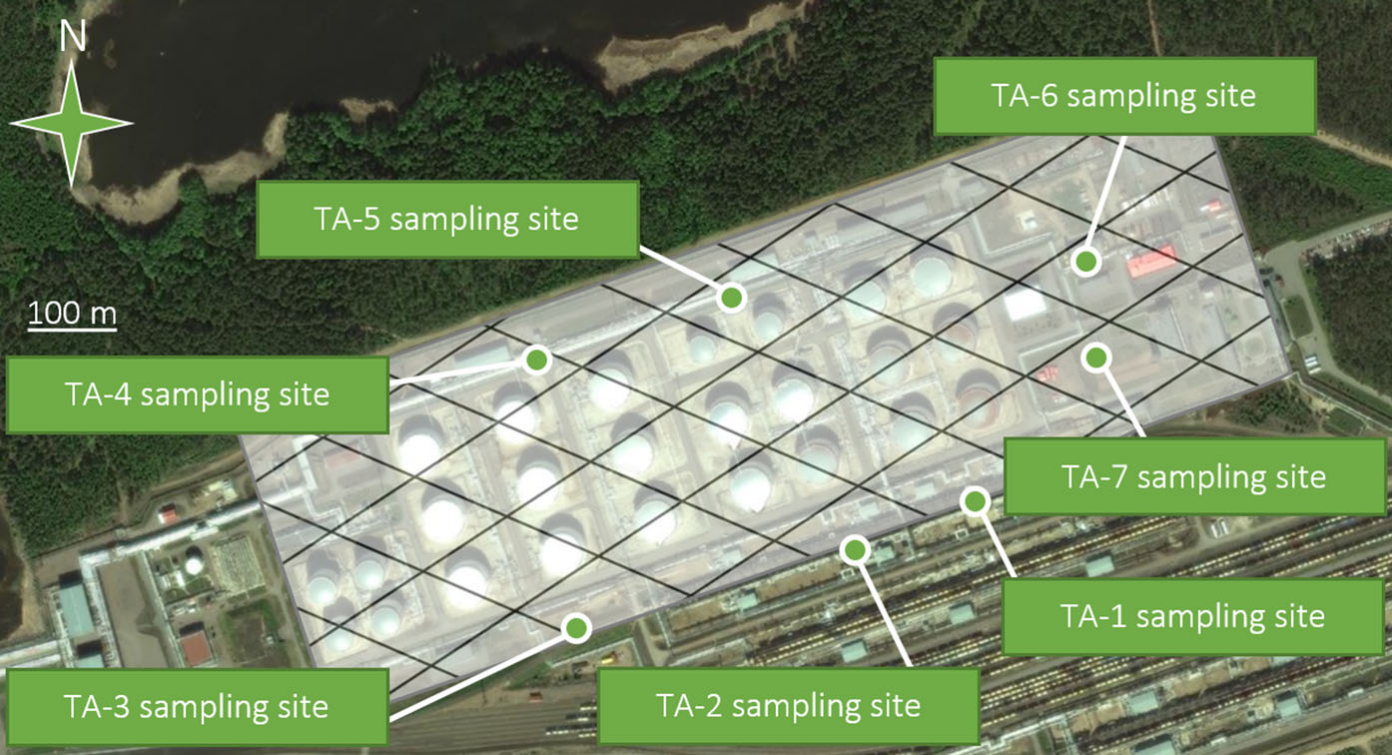
Layout of soil sampling sites in the taiga landscape; the semitransparent diagonal-gridded polygon covers the production site (based on the *Yandex Maps*)

An environmental survey was conducted in the summer of 2017. Basing on visual and organoleptic indicators and taking into account the fact that most of the study area was concreted or paved, potentially contaminated soils were sampled along the perimeter of the tank farm. The location of the test sites is shown in Fig. 5. To comprehensively study the transformations caused by petroleum products with depth, samples were taken from the 3 horizons at each of the 7 test sites with a total number of 21 combined soil samples:

– *A0*, a forest leaf litter topsoil layer, 0–5 cm;

– *A1*, a humus-accumulative horizon, 5–15 cm; and.

– *A2*, an eluvial horizon, 15–20 cm.

A production site of a large mining enterprise was *the third study object* where soils were polluted at a mechanical transport park of quarry equipment. The territory of the production facility belongs to the forest steppe landscape zone: the climate is moderately warm, moderately humid, precipitation amount is equal to moisture evaporation, but droughts are possible. The soils of the land plot of the studied production facility are gray forest, rich of humus and fertile (Naumov 2016), recognized worldwide as Phaeozems (IUSS 2014).

**Fig. 6:**
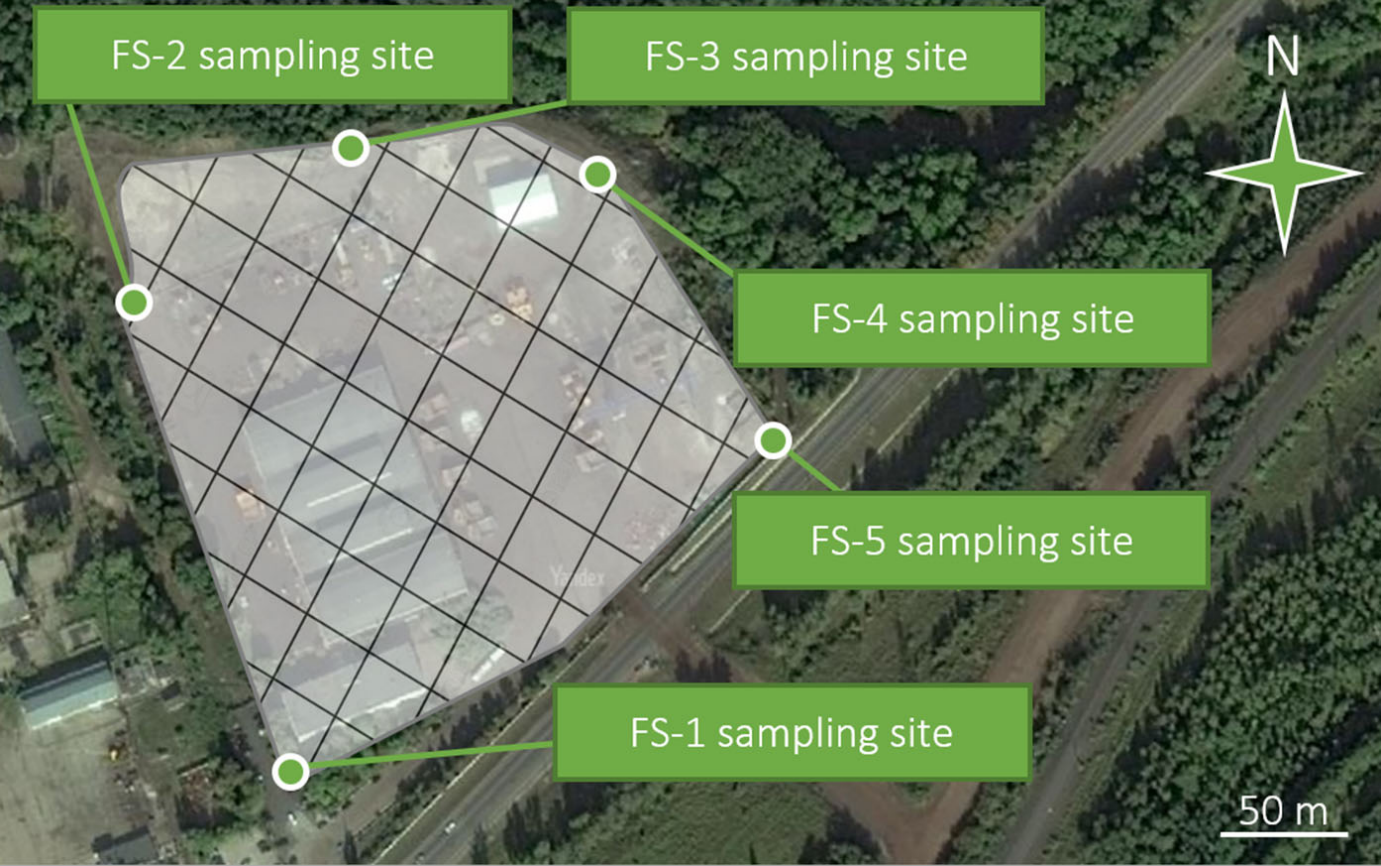
Layout of soil sampling sites in the forest steppe landscape; the semitransparent diagonal-gridded polygon covers the production site (based on the *Yandex Maps*)

To consistently study the territory in a similar way, an environmental survey was conducted in the summer of 2018 and 5 test sites of 1 × 1 m were chosen (Fig. 6.) along the perimeter of the fenced territory of the car park of quarry equipment following the same steps specified above.

### *Laboratory analyses and *ex situ* decontamination experiments*

Determination of the gross content of petroleum products in soil samples was carried out using a standard method for measuring the mass fraction of petroleum products in soil samples by means of the fluorometric method using the *Fluorat-02* liquid analyzer (Lumex, Russia). To obtain trustworthy results, the gross content of oil products in soil samples was determined after the extraction of petroleum products by means of the Fourier-transform infrared spectroscopy using the *IRAffinity-1* spectrometer (Shimadzu, Japan). Moisture content in the samples was measured using the *MX-50* humidity analyzer (A&D, Japan). The total organic carbon content was determined using the *TOC-V CSH* analyzer (Shimadzu, Japan). All analytical procedures were performed in the Common Use Center of the Saint Petersburg Mining University.

To date, the *Procedure for determining the extent of damage from chemical soil contamination* is the baseline document that recommends the threshold levels of concentration of petroleum products in soils of the Russian Federation (Table 1). These figures were applied as an assessment scale as well as a criterion of the necessity for the consequent soil remediation.

The required conditions for thermal desorption cleaning of soils from petroleum products were provided using the *LECO TGA-701* thermogravimetric analyzer (Leco Corporation, USA). We used this equipment to determine the humidity, ash content, and amount of volatile substances in organic, inorganic and synthetic materials when heated. The analyzer was used directly to establish the necessary working temperature range due to the fact that the purpose of the experiment at this research point was to establish the optimal regime at which the concentration of petroleum products in the soil decreases to an acceptable level.

## Results and discussion

### Contamination levels and primary soil quality assessment

The results obtained in the contaminated tundra landscape are presented in Table 2. According to the classification of the level of soil contamination with petroleum products, it can be concluded that the contamination level of the major part of the studied soils is critical (over 5,000 mg/kg). More than a 100-fold exceedance over the permissible level indicates the formation of stable emulsions of petroleum products with water and their accumulation in a cold climate and high Gleysol humidity.

**Table 2:** Concentrations of petroleum hydrocarbons in soil samples from the contaminated tundra landscape

Sampling sites	Petroleum products content, mg/kg	Contamination level
TU-1	16,500	Critical
TU-2	372,500	Critical
TU-3	41,500	Critical
TU-4	650	Allowable
TU-5	4800	High
TU-6	311,000	Critical

**Fig. 7:**
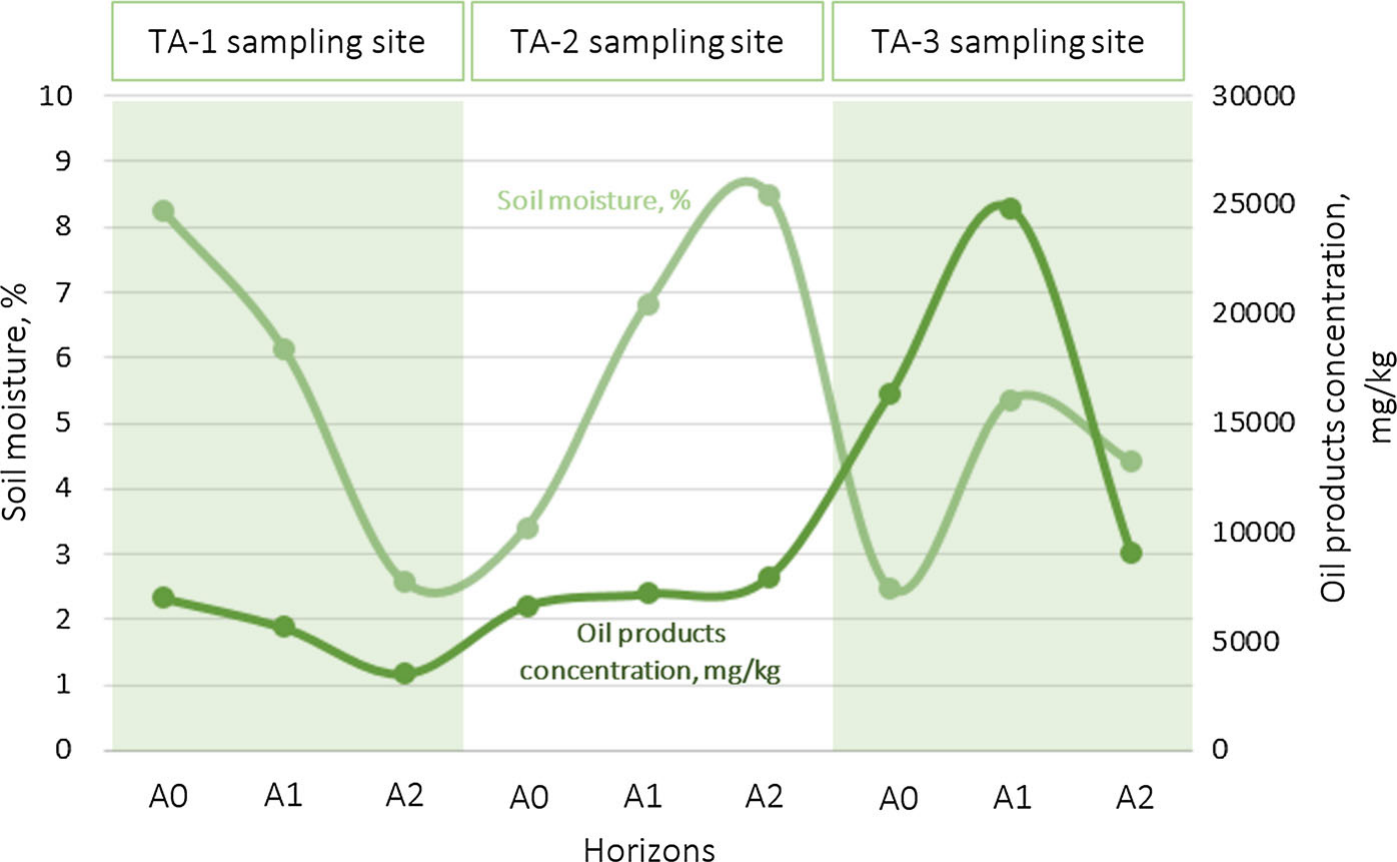
Soil moisture and petroleum hydrocarbon content in the spaces between particles in different horizons

The obtained data on humidity and concentrations of petroleum products in the Podzols are set out in Table 3. Comparing the results with the threshold concentrations, it was found that concentrations in 9 soil samples (1–3 test site) out of 21 exceed the permissible level of petroleum products (1,000 mg/kg). Thus, these samples are highly and critically contaminated. We compared the moisture content and total content of petroleum products in samples with the highest concentrations exceeding the threshold. It was found that the higher the soil moisture, the higher the possible content of petroleum products in it, i.e., the statement that it is possible to form stable lithogeochemical plumes of water–oil compounds, was confirmed. The correlation of values can be observed in Fig. 7. It should be noted that the link between the concentration of petroleum hydrocarbons and soil moisture is most clearly visible within each separately considered sampling site.

**Table 3:** Basic properties of oil-contaminated soils from the taiga landscape

Sampling sites	Soil horizons	Moisture content, %	Petroleum products content, mg/kg	Contamination level
TA-1	A0	8.23	7000	Critical
	A1	6.12	5650	Critical
	A2	2.57	3540	High
TA-2	A0	3.41	6650	Critical
	A1	6.82	7200	Critical
	A2	8.50	7950	Critical
TA-3	A0	2.49	16,300	Critical
	A1	5.36	24,850	Critical
	A2	4.42	9100	Critical
TA-4	A0	6.28	80	Allowable
	A1	6.57	50	Allowable
	A2	5.82	60	Allowable
TA-5	A0	6.03	150	Allowable
	A1	7.48	290	Allowable
	A2	7.07	50	Allowable
TA-6	A0	23.44	30	Allowable
	A1	12.10	20	Allowable
	A2	8.21	10	Allowable
TA-7	A0	1.63	30	Allowable
	A1	3.98	40	Allowable
	A2	2.68	30	Allowable

The results of the forest steppe landscape study are given in Table 4. Assessment of the concentrations revealed that the permissible level is exceeded in 3 soil samples out of 5. The maximum concentration value is 8000 mg/kg, which presumably confirms contamination of the soils adjacent to the production site as a result of the flushing of petroleum products by storm drains.

**Table 4:** Concentrations of petroleum hydrocarbons in soil samples from the contaminated forest steppe landscape

Sampling sites	Petroleum products content, mg/kg	Contamination level
SF-1	351	Allowable
SF-2	604	Allowable
SF-3	2190	Medium
SF-4	8000	Critical
SF-5	2060	Medium

**Fig. 8:**
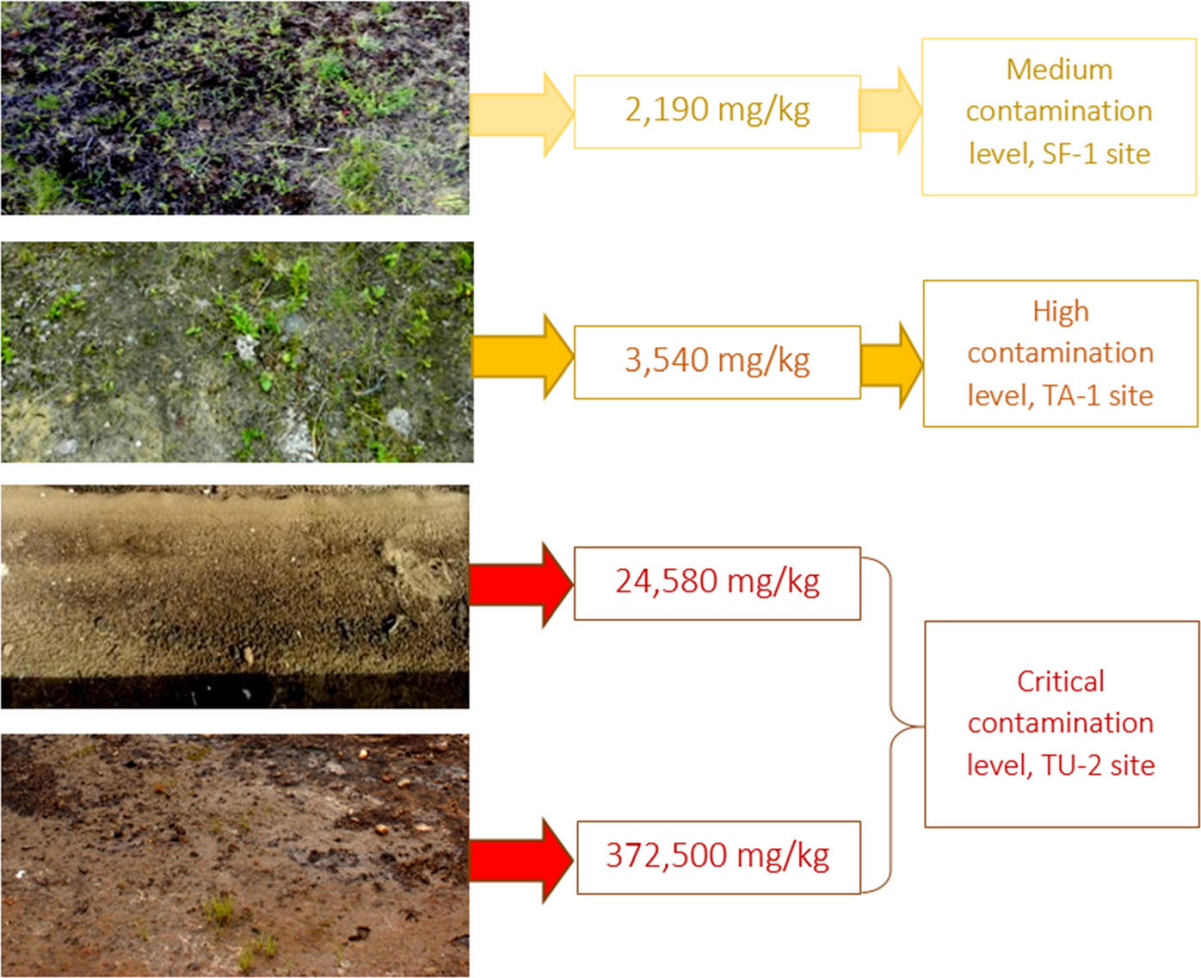
Visually assessed plant cover state and analytically determined contamination levels of soils in the tundra, taiga, and forest steppe landscapes

Comparing the results of field assessment of all the studied industrial areas with the outcomes of laboratory-determined concentration of oil products in soils, it was proved that inhibition of vegetation manifests itself to one degree or another when either the permissible content (1000 mg/kg) is exceeded by tens or hundreds of times (up to 372,500 mg/kg), or in the case of a relatively slight excess at a concentration of 2190 mg/kg (Fig. 8). In this case, we can say that even under the minor technogenic load of oil products on the soil, the significant effects on plant productivity are possible.

Based on the presented results of assessing the level of soil contamination with petroleum products, we can conclude that the development of a remediation method remains relevant. To date, there are many approaches to ex situ desorption of hydrocarbon pollutants from soils, but there is no universal technique.

### Determining optimal regimes of thermal desorption treatment

The first series of experiments were carried out with 9 oil-contaminated soil samples taken from the territory of the reservoir park in the boreal forest zone. The treatment was performed under conditions of maximum rapid heating (according to the equipment specification) in the temperature range of 25–170 °C at the moderate oxygen consumption level. However, after determining the residual content of petroleum products in the treated soils, we found no reduction to the acceptable level. The subsequent increase in the final processing temperature to 200 °C reduced the residual concentration of petroleum products by 7 times, but also did not allow to achieve the desired result.

**Fig. 9:**
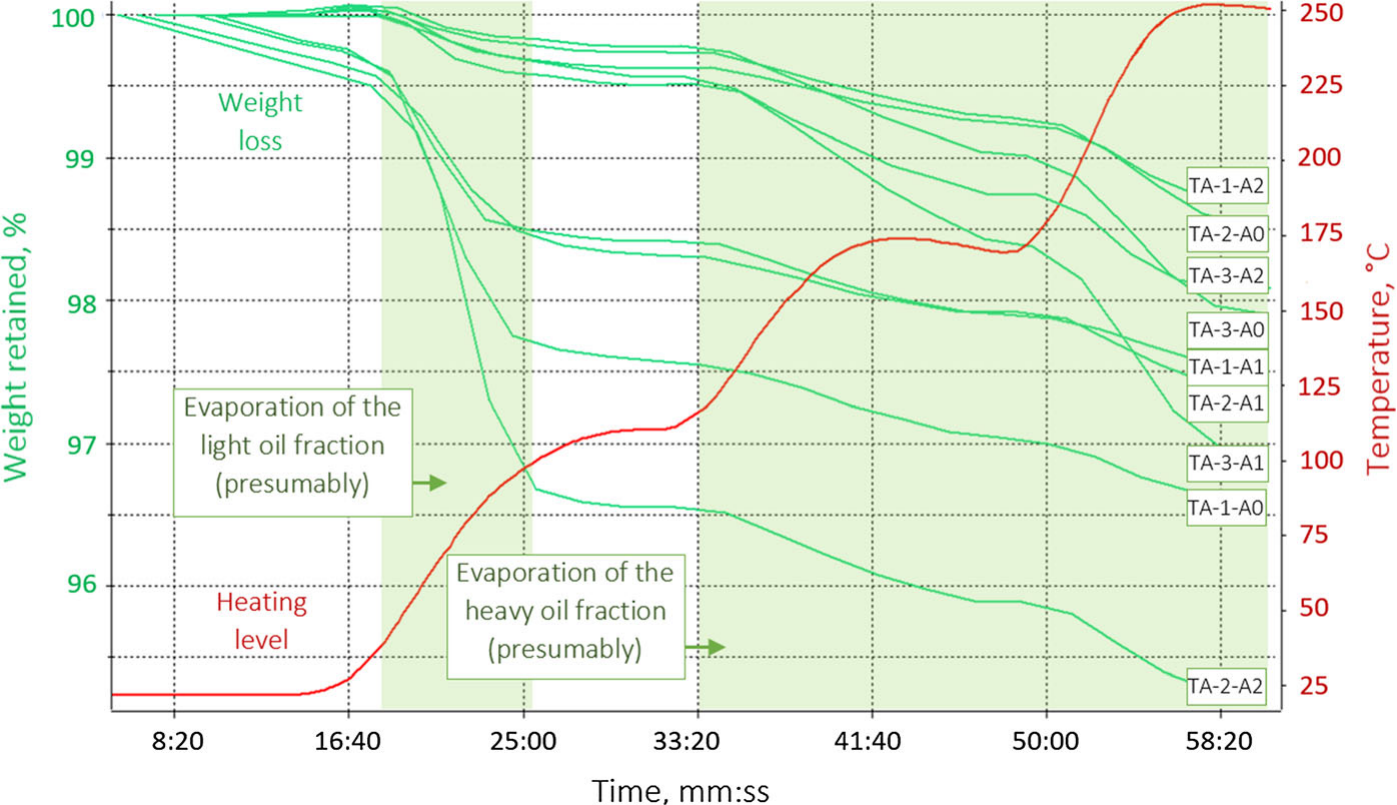
Thermogravimetric analysis results determining the optimal operational temperature regime for remediating the oil-contaminated soils in the taiga landscape

After a series of experiments, the optimal temperature of thermal desorption treatment was established as reflected in Fig. 9. As can be seen from the presented thermogravimetric curve, the final processing temperature is 250 °C, which is only 55% of the burning temperature of all humates. At the same time, the concentration of petroleum products in polluted soils was reduced to concentrations significantly lower than the permissible level (Table 5).

**Table 5:** Concentrations of petroleum hydrocarbons before and after heat treatment of soil samples from the tank farm in the taiga landscape

Sampling sites	Soil horizons	Petroleum products content in soils before treatment, mg/kg	Petroleum products content in the remediated soils, mg/kg	Contamination level
TA-1	A0	7000	0	Allowable
	A1	5650	0	
	A2	3540	0	
TA-2	A0	6650	360	
	A1	7200	410	
	A2	7950	130	
TA-3	A0	16,300	20	
	A1	24,850	1	
	A2	9100	1	

Analyzing the obtained results, it was assumed that despite the low processing temperature, thermal desorption of petroleum products occurs due to the fact that when heating petroleum products in a mixture, the desorption temperature of some components of this mixture may be significantly lower than the evaporation temperature of each component (Akhmetov, 2002).

Humus content was studied to estimate the residual fertility of thermally treated soils. We used one of the indirect methods for determining the total amount of humus, namely, the calculation of its content by the amount of organic carbon. The average carbon content in humus is 58%, and its total amount in the soil can be calculated by multiplying the percentage of organic carbon in soil by a factor of 1.724. Although this factor is conditional and gives only an approximate knowledge of the total amount of humus, at the moment there are no direct and accurate methods for humus determination.

The studied soils are classified as transitional between Cryptopodzols and Mesopodzols (IUSS 2014), as sands and sandy loams are the dominant parent rocks, the humus content in this type of soil typically varies from 2 to 4% in an undisturbed state. The results of determining the total organic carbon and the approximate amount of residual humus are presented in Table 6. As seen from the presented results, the residual humus content varies from 0.4 to 1.5% and the average value is 0.8%. The presence of humus in thermally treated soils shows that no critical destructive changes have occurred, which indicates the possibility of reusing these soils for reclamation, provided that the necessary amount of fertilizer is applied.

**Table 6:** Results of organic carbon analysis and calculation of the humus content

Sampling sites	Soil horizons	Organic carbon content, %	Humus content,%
TU-1	A0	0.309	0.5
	A1	0.543	0.9
	A2	0.444	0.8
TU-2	A0	0.804	1.4
	A1	0.867	1.5
	A2	0.554	0.9
TU-3	A0	0.210	0.4
	A1	0.198	0.3
	A2	0.215	0.4

The established temperature regime was successfully applied for cleaning the soil from oil products taken from the territory of the car park of quarry equipment in the forest steppe landscape, as can be seen from the results presented in Table 7. The residual levels of petroleum products meet the state quality standard requirements and bring an opportunity to utilize the treated soil when revitalizing the industrial brownfield.

**Table 7:** Concentration of petroleum products before and after heat treatment of soil samples from the mechanical transport park of quarry equipment in the forest steppe landscape

Sampling sites	Petroleum products content in soils before treatment, mg/kg	Petroleum products content in the remediated soils, mg/kg	Contamination level of the remediated soils
SF-1	2190	415	Allowable
SF-2	8000	670	
SF-3	2060	345	

## Conclusions

Engineering and environmental survey of the territories of several extraction and processing facilities revealed a significant anthropogenic load on the soil cover. Local technological leaks and accidental spills can lead to the formation of lithogeochemical contamination plumes and flows. It was found that the level of pollution in the territories of the studied objects is 2–25 times higher than the permissible content of petroleum products of 1000 mg/kg. In each case, the oil content of the soil may be affected by many factors such as climatic zone, the volume of petroleum products that gets into the soil, and the type of hydrocarbons.

The optimal operational temperature regime from 25 to 250 °C was established experimentally, allowing the reduction of petroleum products concentrations to an acceptable level, while the final processing temperature is significantly lower than the combustion point of all humates (450 °C). Positive remediation results were obtained for soils with a concentration of petroleum products in a wide range (from 2000 to 25,000 mg/kg). The conducted experimental studies will be used in the future to develop an engineering solution for the implementation of thermal desorption treatment.

Oil-contaminated Gleysols, Podzols, and Phaeozems can be considered as Technosols, i.e., soils that have been changed, moved or formed as a result of human engineering and economic activity. On the contrary, the remediated soil can be reused by enterprises for filling in territories or for land reclamation with the condition of applying the necessary amount of fertilizers. This approach can have an economic effect, which is manifested in the absence of the need to transfer oil-contaminated soils for disposal by third-party organizations and the purchase of clean soil.

## Data Availability

Not applicable.
